# THE TIPPING POINT STUDY: DIGITAL DETECTION AND DECISION SUPPORT FOR OLDER ADULTS AND FAMILIES

**DOI:** 10.1093/geroni/igz038.2217

**Published:** 2019-11-08

**Authors:** Kimberly D Shea, Kimberly D Shea

**Affiliations:** University of Arizona, Tucson, Arizona, United States

## Abstract

In 10 years, the United States will experience a “dependency” ratio of one working age adult (20-64 years old) to one non-working person (> 65 or 85 years old will comprise 19 million of the non-working people (US Census Bureau, 2008). Busy working adults will have to be vigilant to determine when to make life-changing decisions about health and safety issues for people that depend on them. Older adults have gradual and cumulative physical and/or psychological aging changes or can experience significant events. Knowing when to make a life-changing decision, such as when to intervene with independent living due to safety risks, is difficult even when situations have constant vigilance. Eventually, older adults experience a seemingly abrupt, sudden and absolute point where a life changing decision must be made. This is the Tipping Point. Health data, derived from unobtrusive wearable sensors, are algorithmically synthesized to provide critical information on impending concerns via an electronic portal will help the busy working adult to predict and prevent the Tipping Point. This application of precision health care results in targeted and personalized education thus avoiding a potentially catastrophic Tipping Point. This symposium provides insight into five aspects of the Tipping Point: 1) significance of identification, 2) theoretical foundation for environmental and cultural sensitivity, 3) feasibility outcomes from a Mexican American population, 4) methodology for synthesizing quantitative metrics from multivariate streams of data, 5) creation of a culturally sensitive electronic portal to display predictive information and education about consequences

